# The prevalence of liver abnormalities in humans due to *Schistosoma japonicum* by ultrasonography in China: a meta-analysis

**DOI:** 10.1186/s12879-022-07241-5

**Published:** 2022-03-08

**Authors:** Man-Man Gu, Meng-Tao Sun, Jie-Ying Zhang, Qiu-Fu Yu, Da-Bing Lu

**Affiliations:** Department of Epidemiology and Statistics, School of Public Health, Soochow University, Suzhou, China

**Keywords:** *Schistosoma japonicum*, Liver abnormalities, Ultrasonography, Disease burden

## Abstract

**Background:**

*Schistosoma japonicum* was once one of the most severe parasitic diseases in China. After 70 years of national schistosomiasis control programmes, the prevalence and associated morbidity of the infection have been reduced to a much lower level. However, due to the low sensitivity of the current detection approaches, many minor infections in humans could not be identified and ultimately develop chronic injuries with liver abnormalities, a specific ‘network’ echogenic pattern under ultrasonography. Therefore, as more people take part in physical examinations, we performed this meta-analysis to estimate the overall prevalence of schistosomiasis-associated liver abnormalities in China.

**Methods:**

The publications were searched systematically across five electronic databases. All eligible studies were assessed with quality evaluation forms. Heterogeneity of studies was determined using the I^2^ and Q tests. A random effects or fixed effects model was employed based on heterogeneity results. The pooled prevalence and its 95% confidence intervals were calculated with the Freeman-Tukey double arcsine transformation. All analyses were conducted using R with the “meta” package. The protocol registration number was CRD42021232982.

**Results:**

A total of 19 relevant articles, including 21 studies, were included. The average score of study quality was 6.4 (total score 7), indicating high quality of all included studies. A total of 268, 247 persons were included, and 43, 917 persons were diagnosed with schistosomiasis liver abnormalities by ultrasonography. High degrees of heterogeneity existed among all studies or within subgroups. The overall pooled prevalence was 18.64% (95% CI: 11.88–26.50%). The estimate significantly increased over time and varied among provinces, with the highest in Shanghai and the lowest in Sichuan. The estimate in people aged 60 years or older was significantly higher than that in people of all ages. No significant difference was seen when based on study areas (urban or rural areas) or gender.

**Conclusion:**

The long-term burden of schistosomiasis in China remains large, as nearly one-fifth of the examined persons were diagnosed with schistosomiasis liver abnormalities. The pooled prevalence was associated with regions or age groups. Such may have a high reference value in the exact calculation of the disease burden and can be helpful for policy makers in prioritizing public health.

**Supplementary Information:**

The online version contains supplementary material available at 10.1186/s12879-022-07241-5.

## Background

Schistosomiasis is primarily endemic in tropical and subtropical areas and is typically associated with poor environmental and sanitary conditions [1, 2]. This infectious disease affects nearly 240 million people worldwide, with more than 700 million people at risk of infection. Infections occur when people encounter freshwater contaminated by schistosome cercariae. The cercariae penetrate skin and invade the human body. They live as adult worms in the veins draining the urinary tract (*S. haematobium*) or intestine (*S. mansoni*, *S. japonicum*), with male and females pairing up. A lot of eggs produced by schistosome females accumulate in tissues, which causes great damage to the body [1, 3]. Infected individuals with acute or chronic morbidity manifest as two types: urinary schistosomiasis caused by *Schistosoma haematobium* and intestinal schistosomiasis mainly caused by *S. japonicum* and *S. mansoni*. For intestinal schistosomiasis, as schistosome eggs are transported to the liver through the mesenteric circulation, granulomas and portal fibrosis in the liver will develop due to the host's immune response, thus leading to schistosome-associated liver abnormalities [4, 5]. With an estimate of at least 236.6 million people requiring preventive treatment in 2019 (https://www.who.int/news-room/fact-sheets/detail/schistosomiasis), schistosomiasis would be a major cause of liver fibrosis in areas where schistosomiasis is endemic [6–8].

*S. japonicum* was once one of the most severe parasitic diseases in China. After nearly 70 years of multistage comprehensive prevention and control, the prevalence and associated morbidity of the infection has been reduced significantly in many endemic areas [9, 10]. However, the burden caused by chronic damage to the disease is still huge. In 2017, for example, the disability-adjusted life years (DALYs) of patients with advanced schistosomiasis in China were 15, 679.02 person-years, accounting for 73.24% of the total disease burden [11]. As quite a number of minor infections in humans could not be detected, coupled with clinical unawareness [12], the disease burden in China might have been greatly underestimated. This is typically evident from the official report [13], in which approximately one thousand cases of advanced schistosomiasis have been reported each year.

PZQ treatment has recently been shown to induce partial or complete regression of periportal fibrosis in early fibrosis [14–18], indicating that the morbidity of schistosomiasis-related liver abnormalities could be preventable through early treatment. At present, ultrasound technology, due to its fast, accurate, and noninvasive characteristics in physical examination, has been widely used in all clinical practice. Schistosomiasis-related liver abnormalities show a specific ‘network’ echogenic pattern, significantly different from other liver diseases such as cirrhosis [18, 19]. With socioeconomic development in China, an increasing number of people take part in annual or regular physical examinations, in which ultrasound techniques have been routinely used. Therefore, we performed this meta-analysis to estimate the overall prevalence of schistosomiasis liver abnormalities in China when the country has been moving onto disease elimination and to investigate any differences among regions or populations. The results from this study would, apart from prioritizing public health improvement on the people affected, be helpful in accurately estimating the long-term burden of the disease.

## Methods

### Search strategy and selection criteria

This meta-analysis was based on the Preferred Reporting Items for Systematic Review and Meta-Analyses (PRISMA) guidelines [20] (Additional file 1) with its protocol registration number (No. CRD42021232982). The index of interest for this article is the overall prevalence of schistosomiasis liver abnormalities in humans in China. Therefore, we searched all relevant documents published before February 1, 2021. The selection of publications was performed systematically in the five electronic databases CNKI, VIP, and Wanfang (in Chinese) and PubMed and Web of Science (in English). The terms we used and combined in the search included “ribenxuexichong and/or xuexichongganbing and tijian and chaoshengjiancha” (pinyin in Chinese) and “*Schistosoma japonicum* and schistosomiasis and liver abnormalities and ultrasonography” (in English). In addition, we manually searched for more relevant literature in the references of the retrieved articles.

Two independent researchers (GM and MS) extracted the data into an Excel form. Any differences in the extracted data were discussed with a third researcher (YQ) until a consensus was reached. The preliminary review of the title and abstract of the retrieved documents were conducted by GM and MS. The duplicated and the records with the abstract only were removed. Eligible publications were screened by full text articles reading. The inclusion criteria for all articles were as follows: (1) conducted in epidemic areas of China; (2) about S. japonicum-associated liver abnormalities; (3) diagnosed by ultrasonography with the specific image of grade II or above [19, 21], a specific ‘network’ echogenic pattern; (4) prevalence or numbers of investigated and inflicted persons reported; (5) populations participating in a physical examination; and (6) including data on the following outcome of interest (if possible): time performed, location and age groups of subjects. There were no restrictions on age and region. All studies conducted outside endemic areas of China, involving physical examination without using ultrasonography, and reporting outcomings other than liver abnormalities were excluded.

### Data extraction and quality appraisal

For eligible articles, the data to be extracted included the following: the first author, year of articles published, year of research performed, regions, study setting (urban or rural areas), target populations (gender and age), the total number of people who participated in the physical examination and the number of people with schistosomiasis liver abnormalities.

All eligible articles were evaluated for quality. To be in line with this research, our evaluation tool was modified based on the Joanna Briggs Institute Prevalence Critical Appraisal Tool [22, 23]. All selected studies were assessed using seven quality control items (below), and for each item, two options were given, i.e., yes and no. "Yes" means that the standard was met and a score of 1 point was given; otherwise, "No" and 0 points were given. The score of a study ranged from 0 to 7 points. A study with 3 points or less was considered low quality, 4 to 5 points medium quality, and 6 to 7 points high quality. Only articles with medium and high quality were included in this meta-analysis. Two evaluators independently scored the quality of each included article. The seven quality control items used were:

A. representativeness of the sample to the target population.
B. appropriateness of the method used to sample study participants.
C. adequateness of sample size.
D. description of study subjects and settings.
E. validity of the method (ultrasound diagnostic technique) used.
F. clarity of prevalence data or data can be calculated for all participants.
G. adequateness of prevalence rate.

### Data analysis

All the extracted data were summarized in an Excel form and were then imported into R version 4.0.4 and R studio with the “meta” package for statistical analysis. For each study, the prevalence of schistosomiasis liver abnormalities was first transformed with Freeman-Tukey double arcsine [24–26]. The pooled prevalence and its 95% confidence intervals (CIs) were then obtained by summarizing the results of all studies based on the accuracy of each study- "weight" [24]. The inverse variance statistic (*I*^2^ index) was used to test the quantity of heterogeneity, and the Cochran Q test was used to determine whether the difference was statistically significant at the significance level of 0.05. The *I*^2^ index was interpreted as no, low, moderate, or high heterogeneity if the index was 0, ≤ 25%, ≤ 50%, or ≤ 75%, respectively [27–29]. When the heterogeneity was not obvious (≤ 50%), the pooled prevalence was calculated using a fixed effects model; otherwise, a random effects model was used [28].

Subgroup analysis was performed to explore the factors that might contribute to the source of heterogeneity. These included the study period, setting (urban or rural areas), regions (at the province level), gender and age groups. The prevalence and 95% CIs of each study and group were drawn in forest plots. Publication bias was evaluated through visual inspection of funnel plots and then was statistically evaluated with Egger’s test [30, 31].

## Results

### Search results and study selection

**Fig. 1:**
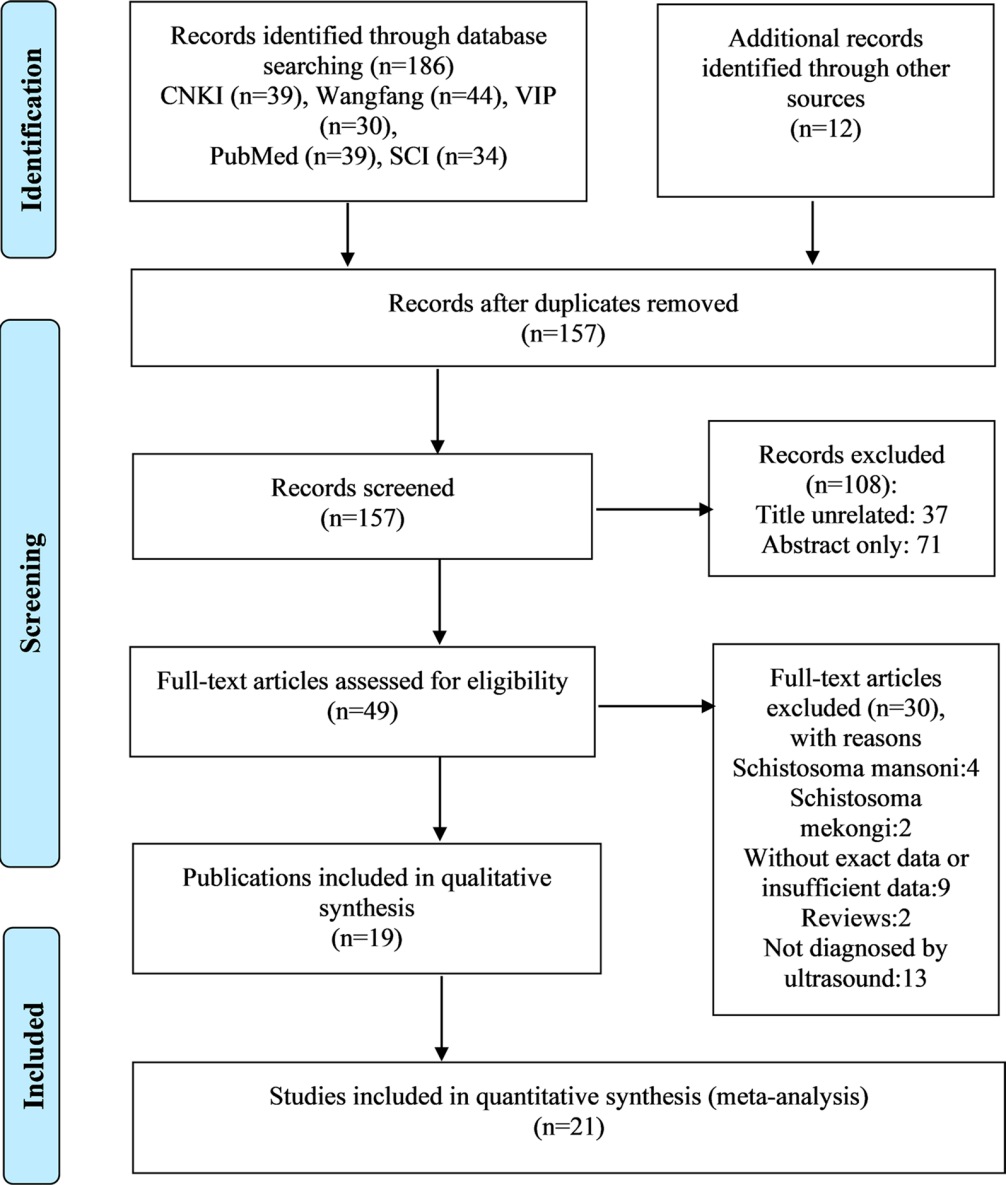
Flow chart of study selection. The flow diagram shows the numbers of titles and studies reviewed in preparation for this meta-analysis

The workflow is shown in Fig. 1. A total of 198 records were obtained from the five electronic databases. After removing duplicates and carrying out the preliminary screening of abstracts, we retrieved 49 publications with full texts. Of these, a total of 30 unqualified publications were further excluded for the following reasons: four about *S. mansoni*, two about *S. mekongi*, nine without exact data or insufficient data, two reviews, and 13 not diagnosed by ultrasound examination. Finally, a total of 19 articles [32–50] with 21 studies were included in this meta-analysis.

### Study characteristics and quality assessment

The main characteristics of all studies are summarized in Table 1. The 19 articles were conducted in four provinces of China, including nine in Shanghai, four in Jiangsu, four in Zhejiang, and two in Sichuan. Among all included studies, four were conducted before 2010, 11 during 2011 to 2015 and six during 2016 to 2020. A total of 268, 247 persons were included in this analysis, and among them, 43, 917 persons were diagnosed with schistosomiasis liver abnormalities. The prevalence of liver abnormalities in humans among studies ranged from 0.07% to 85.16%. Study quality and risk of bias assessment results showed an average score of 6.4, indicating high quality for all included studies (Additional file 2).

**Table 1:** Characteristics of eligible studies in systematic review and meta-analysis

Author, year	Year of study performed	No examined	Region	Study setting	Age	Liver abnormalities	
						No	%
Xu, 2020 [32]	2017.1–12	3694	Sijin, Shanghai	Urban	≥ 65	1359	36.79
Jiang, 2020 [33]	–	869	Jinshan, Shanghai	Urban	≥ 60	740	85.16
Wang, 2019 [34]	2015	528	Suzhou, Jiangsu	Rural	≥ 65	310	58.70
Gu, 2019 [35]	2017	4950	Songjiang, Shanghai	Urban	≥ 65	201	4.05
Huang, 2018 [36]	2011–2016	22,720	–	–	–	132	0.58
Jiang, 2018 [37]	2016	6940	Songjiang, Shanghai	Urban	≥ 65	2122	30.58
Jin, 2015 [38]	–	2027	Fengxian, Shanghai	Rural	> 65	1069	52.70
Fu, 2014 [39]	2013	1393	–	–	–	8	0.57
Xu, 2014 [40]	2012	7800	Suzhou, Jiangsu	Urban	≥ 65	1777	22.78
Xia, 2013 [41]	2011–2012	11,666	Shaoxing, Zhejiang	Rural	Mean 58.6 ± 6.2	22	0.19
Zhou, 2013 [42]	2012.6–7	3092	Songjiang, Shanghai	Urban	≥ 65	1577	51.00
Zeng, 2013 [43]	2008	117,979	Minhang, Shanghai	Mixed	≥ 60	13,839	11.73
Xu, 2012 [44]	2005–2011	49,395	Jiading, Shanghai	Rural	≥ 60	16,029	32.45
Zhou, 2012 [45]	2011.5–2012.5	4817	Wujiang, Jiangsu	Mixed	55–90	986	20.47
Mao, 2012 [46]	2009	3397	Jiashan, Zhejiang	Rural	35–65	891	26.23
	2010	6696				1376	20.55
	2011	4016				987	24.58
Gu, 2010 [47]	2009.1	1620	Fengxian, Shanghai	Urban	≥ 60	16	0.99
Yang, 2009 [48]	2006.9–2007.9	8208	Suzhou, Jiangsu	Urban	18–90	422	5.14
Tang, 2008 [49]	2001	657	Guanghan, Sichuan	Rural	5–65	50	7.61
Huang, 2003 [50]	2001.6–7	5783	Chengdu, Sichuan	Urban	–	4	0.07

### Pooling and heterogeneity analysis

**Fig. 2:**
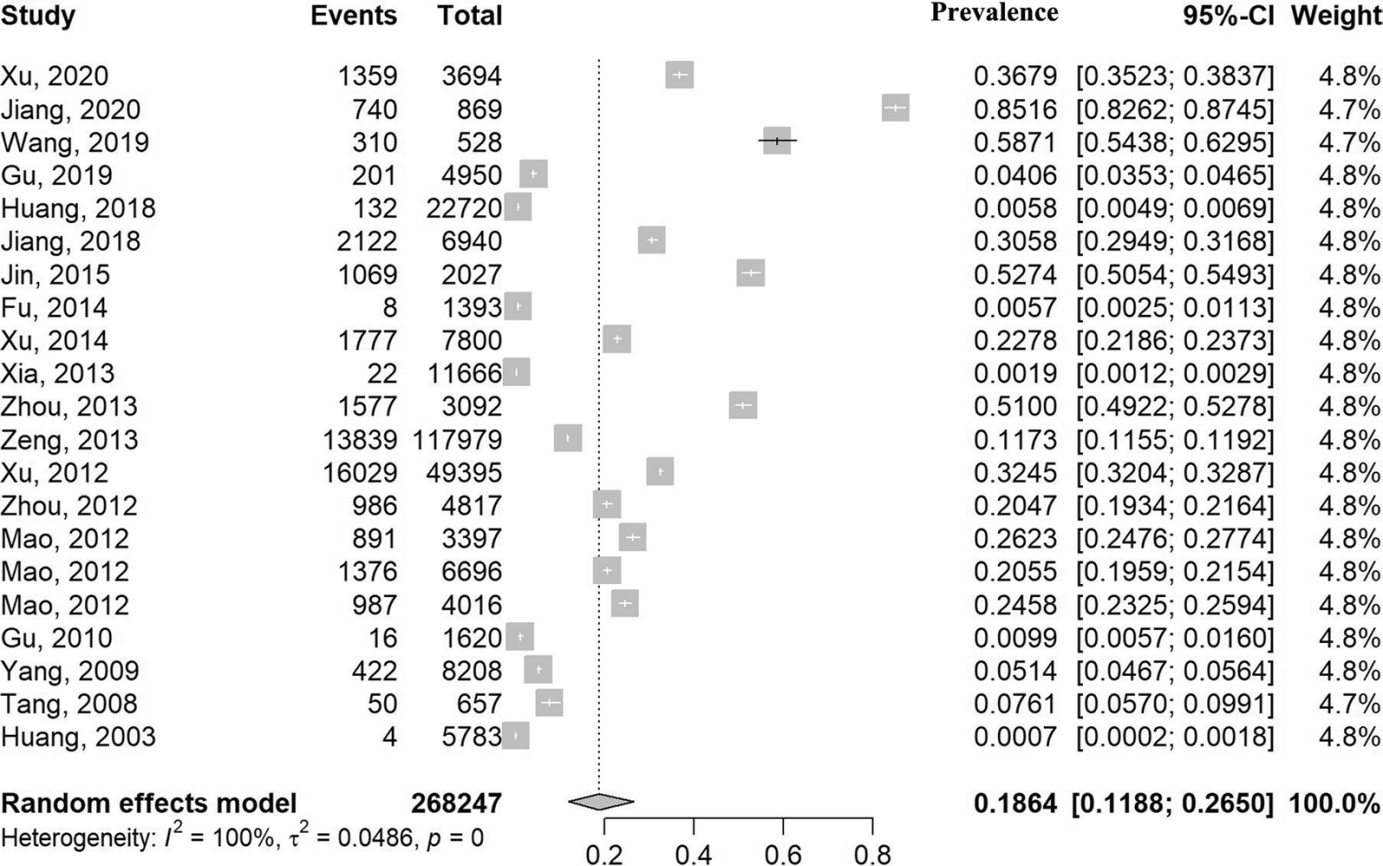
Forest plot and pooled prevalence of schistosomiasis liver abnormalities showing 21 datasets collected from 19 publications. The diamonds delimit the 95% CIs of all individual prevalence and the overall prevalence, estimated using a random effects model. The included studies were ordered by year of publication

**Fig. 3:**
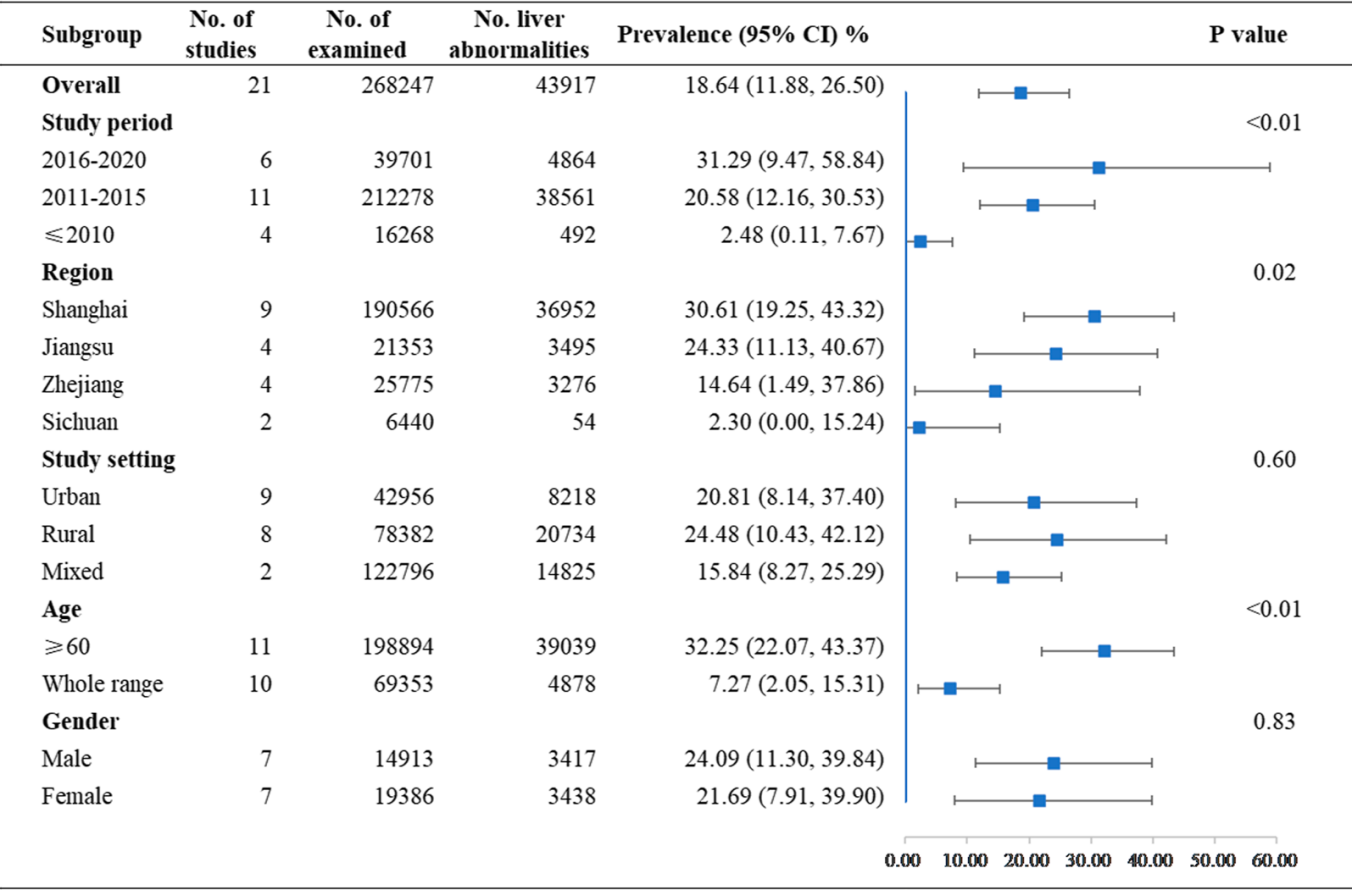
Forest plot of schistosomiasis liver abnormality prevalence in humans pooled by subgroups. The blue square indicates prevalence, and whisker bars indicate its 95% CI

The prevalence and 95% CIs of schistosomiasis liver abnormalities in humans for each study are shown Fig. 2. As a high degree of heterogeneity existed among studies (χ^2^ = 39, 543.36, p < 0.01; *I*^*2*^ = 99.9%, 95% CI: 99.9–100.0%), a random effects model was used to estimate the overall prevalence. The pooled estimate was 18.64% (95% CI: 11.88–26.50%). The results of subgroup meta-analyses are shown in Fig. 3 and Table 2. As high heterogeneity existed within each subgroup, a random effects model was used to estimate all size effects. The estimated prevalence among the three periods of study was significantly different (p < 0.01) and increased over time, with 2.48% (95% CI: 0.11–7.67%) before 2010, (20.58% (95% CI: 12.16–30.53%) from 2011 to 2015 and 31.29% (95% CI: 9.47–58.84%) from 2016 to 2020. There were significant differences among provinces. Shanghai had the highest prevalence of 30.61% (95% CI: 19.25–43.32%), and Sichuan had the lowest prevalence of 2.30% (95% CI: 0.00–15.24%). The prevalence for people aged 60 years or over (32.25%, 95% CI: 22.07–43.37%) was significantly higher (P = 0.01) than that for the whole age range (7.27%, 95% CI: 2.05–15.31%). No significant difference was seen when based on study areas (urban or rural areas) or genders.

**Table 2:** Pooled prevalence of liver abnormalities due to *S. japonicum* in populations or by subgroups with meta-analysis

	No. studies	No. examined	No. liver abnormalities	Prevalence	Heterogeneity			Egger's test			Subgroup difference	
				(95% CI) %	Q-χ^2^	Q-p	I ^2^ (95% CI) %	t	t/df	P	Q-χ^2^	P
Overall	21	268,247	43,917	18.64 (11.88, 26.50)	39,543.36	< 0.01	99.9 (99.9, 100.0)	0.47	19	0.65		
Study period											10.73	< 0.01
2016–2020	6	39,701	4864	31.29 (9.47, 58.84)	12,033.09	< 0.01	100.0	2.77	4	0.05		
2011–2015	11	212,278	38,561	20.58 (12.16, 30.53)	20,077.86	< 0.01	100.0 (99.9, 100.0)	0.61	9	0.56		
≤ 2010	4	16,268	492	2.48 (0.11, 7.67)	609.12	< 0.01	99.5 (99.3, 99.6)	0	2	0.1		
Region											9.7	0.02
Shanghai	9	190,566	36,952	30.61 (19.25, 43.32)	16,985.76	< 0.01	100.0 (99.9, 100.0)	1.27	7	0.23		
Jiangsu	4	21,353	3495	24.33 (11.13, 40.67)	1824.26	< 0.01	99.8 (99.8, 99.9)	1.07	2	0.4		
Zhejiang	4	25,775	3276	14.64 (1.49, 37.86)	5372.3	< 0.01	99.9 (99.9, 100.0)	2.86	2	0.1		
Sichuan	2	6440	54	2.30 (0.00, 15.24)	150.98	< 0.01	99.3 (98.8, 99.6)	–	–	–		
Study setting											1.02	0.6
Urban	9	42,956	8218	20.81 (8.14, 37.40)	11,725.93	< 0.01	99.9	0.98	7	0.36		
Rural	8	78,382	20,734	24.48 (10.43, 42.12)	13,173.26	< 0.01	99.9 (99.9, 100.0)	− 0.31	6	0.76		
Mixed	2	122,796	14,825	15.84 (8.27, 25.29)	266.56	< 0.01	99.6 (99.4, 99.8)	–	–	–		
Age											14.09	< 0.01
≥ 60	11	198,894	39,039	32.25 (22.07, 43.37)	17,473.32	0	99.9	1.54	9	0.16		
Whole range	10	69,353	4878	7.27 (2.05, 15.31)	9880.6	0	99.9	1.64	8	0.14		
Gender											0.05	0.83
Male	7	14,913	3417	24.09 (11.30, 39.84)	2554.25	0	99.8 (99.7, 99.8)	0.4	5	0.71		
Female	7	19,386	3438	21.69 (7.91, 39.90)	4535.49	0	99.9 (99.8, 99.9)	0.93	5	0.39		

*Cl* Confidence interval, *I*^*2*^ Inverse variance index, *Q-P* Cochran’s P value

### Publication bias and sensitivity tests

**Fig. 4:**
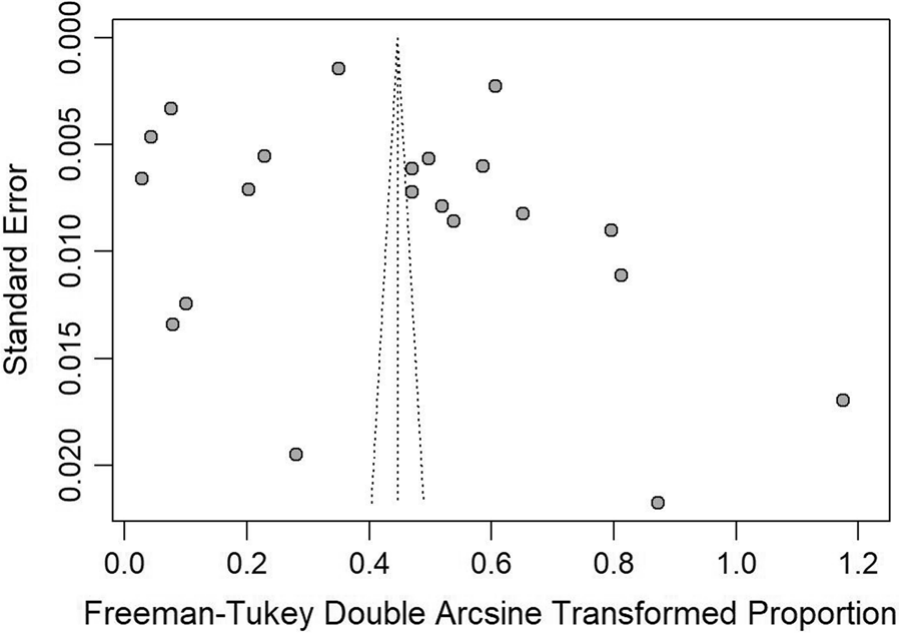
Funnel plots of the Freeman-Tukey double arcsine transformed prevalence of schistosomiasis liver abnormalities. The vertical and diagonal dashed lines represent the overall prevalence and its 95% CI, respectively. Each dot represents a different study

**Fig. 5:**
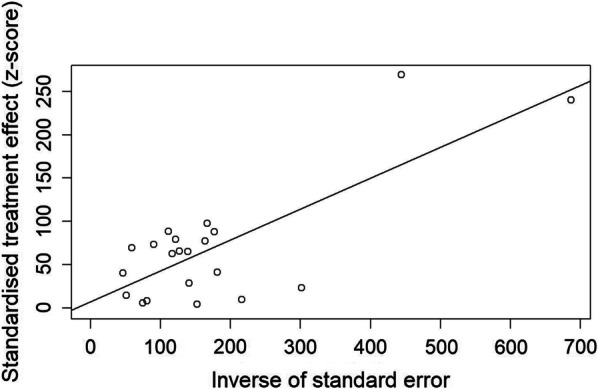
Egger’s publication bias plot of the included studies. The size of circles indicates the sample size of a study

To explore the publication bias among studies, we conducted a funnel plot (Fig. 4) and Egger’s linear regression test (Fig. 5). The results indicated no publication bias (intercept = 0.3565, bias coefficients b = 7.20, t = 0.47, p = 0.65). The sensitivity tests showed that all single-study-omitted estimates were within the 95% CI of the respective overall prevalence (Additional file 3). This suggested that the pooled estimate was not substantially influenced by any single study and hence validated the rationality and reliability of our analyses.

## Discussion

In this meta-analysis of the overall prevalence of schistosomiasis-associated liver damage in humans in China, we retrieved 19 relevant articles involving 21 studies with eligible data from 268, 247 participants, of which 43, 917 were diagnosed with liver abnormalities. The overall pooled prevalence with its 95% CI was 18.64% (11.88–26.50%). Subgroup analyses revealed that the estimate significantly increased over time and varied with regions or age groups of people. Such results would be helpful for health policy makers in both accurately estimating the long-term burden of the disease and setting priorities for life quality improvement on the people affected.

The considerably high prevalence of 18.64% was unexpected but reasonable. Schistosomiasis has been documented in China for more than 2100 years and is one of the most infectious diseases that has seriously affected people's health. For example, it was estimated that in the early 1950s, there were more than 11.6 million patients with schistosomiasis [51]. Although at the end of 2019 the average infection rates in humans and bovines have been reduced to a much lower level (five out of 327, 475 in humans and seven out of 134, 978 in bovines) [52], people with previous infections over last several decades could turn to a great mass. Because minor infections in humans may not be identified with the current diagnostic assays (parasitological or immunological) [53], the number of people with liver abnormalities caused by low-intensity infection (or incomplete treatment) could be large. This is supported by the results of this research. It could suggest that more cases of schistosomiasis liver abnormalities may be undisclosed. Indeed, quite a number of new cases of advanced schistosomiasis have been reported annually, for example a total of 956 in 2019 [13]. Our results did show that the prevalence estimate of liver abnormalities in humans significantly increased over time.

Several factors affected the prevalence estimates. The subgroup analysis by province showed that Shanghai had the highest prevalence (30.61%), followed by Jiangsu (24.33%) and Zhejiang (14.64%) and the lowest Sichuan (2.30%). All these regions were once heavily affected by schistosomiasis, and the elderly people there were very likely to have been infected previously when they were young. Generally, regions with higher economic levels have better public health service and more elderly people will take part in physical examinations, resulting in more cases detected. The prevalence results of this study were, to some extent, in agreement with the reported historical prevalence of schistosomiasis in humans in these regions [54]. Schistosomiasis liver abnormalities usually follow many years of silent or mildly symptomatic infection. Therefore, as expected, the prevalence of liver abnormalities in people 60 years or older was significantly higher than that in those of all ages.

The pooled prevalence of liver abnormalities in rural areas was higher than that in urban areas, and higher in men compared to women, but neither were significant. There is no publication bias in the included literature. We here fully admit the limitations within this study. First, the estimations of prevalence using a random-effects model may not absolutely invalidate the heterogeneity between studies. Overall heterogeneity for pooled and subgroup prevalence estimates was high, suggesting that there was a significant residual effect of unmeasured variables. Second, data paucity exists in the other endemic provinces, mostly with low economic development. This may have underestimated the pooled prevalence in China. Finally, data were missing for gender variables in one-third of the included studies, which made it difficult to estimate the influence of this factor.

We here reported the considerably high prevalence of schistosomiasis-associated liver damage in humans across China, mostly from provinces with schistosomiasis transmission interrupted. However, the results would also have implications for the endemic areas where transmission of the disease has not been interrupted. When the transmission of *S. japonicum* in an area has been reduced to a much lower level and then it is determined whether the criteria for transmission interruption have been met or not, more efforts and resources would be taken in looking for new schistosome infections in humans at young age. The previous infections in humans at old age and particularly their subsequent damage on livers might have been ignored. As the morbidity due to schistosomiasis could be, partially or completely, preventable through early PZQ treatment [14–18], we would recommend that in such areas all old people with previous or suspected schistosome infections receive the treatment of the drug. This may greatly reduce the burden caused by schistosomiasis in the future.

## Conclusions

While China, consistent with the revised WHO global goals, has set the target of the elimination of zoonotic *S. japonicum* transmission by 2030 at the country level [55, 56], the long-term burden of schistosomiasis in China remains huge, as nearly one-fifth (18.64%) of people are diagnosed with schistosomiasis liver abnormalities in our research. The pooled prevalence was associated with study periods, regions or age groups. The results obtained from this study may have a high reference value in the future exact calculation of the DALYs caused by the parasite. They can also be helpful for policy makers to prioritize improving public health for the people with liver damage due to schistosome infections.

## Supplementary Information

**Additional file 1: Table S1.** Preferred reporting items for systematic reviews and meta-analyses checklist.

**Additional file 2: Table S2.** Quality assessment results of all included publications.

**Additional file 3: Fig. S1.** Sensitivity analysis of the pooled prevalence of schistosomiasis liver abnormalities for all studies.

## Data Availability

All data analysed within this study are shown in Table 1.

## Abbreviations

PRISMA: Preferred reporting items for systematic reviews and meta-analyses
DALYs: Disability-adjusted life years. CIs: Confidence intervals
