# Supplementary material for: The prevalence of liver abnormalities in humans due to Schistosoma japonicum by ultrasonography in China: a meta-analysis
Source: BMC Infect Dis. 2022 Mar 8;22:236. doi: 10.1186/s12879-022-07241-5 (PMC8903095; doi:10.1186/s12879-022-07241-5)
Supplement: Supplementary file 2 — Additional file 2: Table S2. Quality assessment results of all included publications. [file 12879_2022_7241_MOESM2_ESM.docx]

Additional file 2: Table S2. Quality assessment results of all included publications.

S2 Table. Quality assessment results

| **SN** | **Author year** | **Quality criteria score** | | | | | | | | | |
| --- | --- | --- | --- | --- | --- | --- | --- | --- | --- | --- | --- |
|  |  | **A** | **B** | **C** | **D** | **E** | **F** | **G** | **Total** | | |
| 1 | Xu, 2020 | 1 | 1 | 1 | 1 | 1 | 1 | 1 | 7 | | |
| 2 | Jiang, 2020 | 1 | 1 | 1 | 1 | 1 | 1 | 1 | 7 | | |
| 3 | Wang, 2019 | 1 | 1 | 1 | 1 | 1 | 1 | 1 | 7 | | |
| 4 | Gu, 2019 | 1 | 1 | 1 | 1 | 1 | 1 | 1 | 7 | | |
| 5 | Huang, 2018 | 1 | 1 | 1 | 1 | 1 | 1 | 0 | 6 | | |
| 6 | Jiang, 2018 | 1 | 1 | 1 | 1 | 1 | 1 | 1 | 7 | | |
| 7 | Jin, 2015 | 1 | 1 | 1 | 1 | 1 | 1 | 1 | 7 | | |
| 8 | Fu, 2014 | 0 | 1 | 1 | 1 | 1 | 1 | 0 | 5 | | |
| 9 | Xu, 2014 | 1 | 1 | 1 | 1 | 1 | 1 | 1 | 7 | | |
| 10 | Xia, 2013 | 0 | 1 | 1 | 1 | 1 | 1 | 0 | 5 | | |
| 11 | Zhou, 2013 | 1 | 1 | 1 | 1 | 1 | 1 | 1 | 7 | | |
| 12 | Zeng, 2013 | 1 | 1 | 1 | 1 | 1 | 1 | 1 | 7 | | |
| 13 | Xu, 2012 | 1 | 1 | 1 | 1 | 1 | 1 | 1 | 7 | | |
| 14 | Zhou, 2012 | 0 | 1 | 1 | 1 | 1 | 1 | 1 | 6 | | |
| 15 | Mao, 2012 | 0 | 1 | 1 | 1 | 1 | 1 | 1 | 6 | | |
| 16 | Gu, 2010 | 1 | 1 | 1 | 1 | 1 | 1 | 0 | 6 | | |
| 17 | Yang, 2009 | 0 | 1 | 1 | 1 | 1 | 1 | 1 | 6 | | |
| 18 | Tang, 2008 | 0 | 1 | 1 | 1 | 1 | 1 | 1 | 6 | | |
| 19 | Huang, 2003 | 0 | 1 | 1 | 1 | 1 | 1 | 0 | 5 | | |
| **Average** | | | | | | | | | | 6.4 |  |

Note:

A-representativeness of the sample to the target population.

B-appropriateness of the way used to sample study participants.

C-adequateness of sample size.

D-description of study subjects and settings.

E-validity of method (ultrasound diagnostic technique) used to diagnose schistosomiasis liver abnormalities.

F-clarity of prevalence data or data can be calculated for all participants.

G-adequateness of prevalence rate.
