# Supplementary figures and images for: The prevalence of liver abnormalities in humans due to Schistosoma japonicum by ultrasonography in China: a meta-analysis
Source: BMC Infect Dis. 2022 Mar 8;22:236. doi: 10.1186/s12879-022-07241-5 (PMC8903095; doi:10.1186/s12879-022-07241-5)

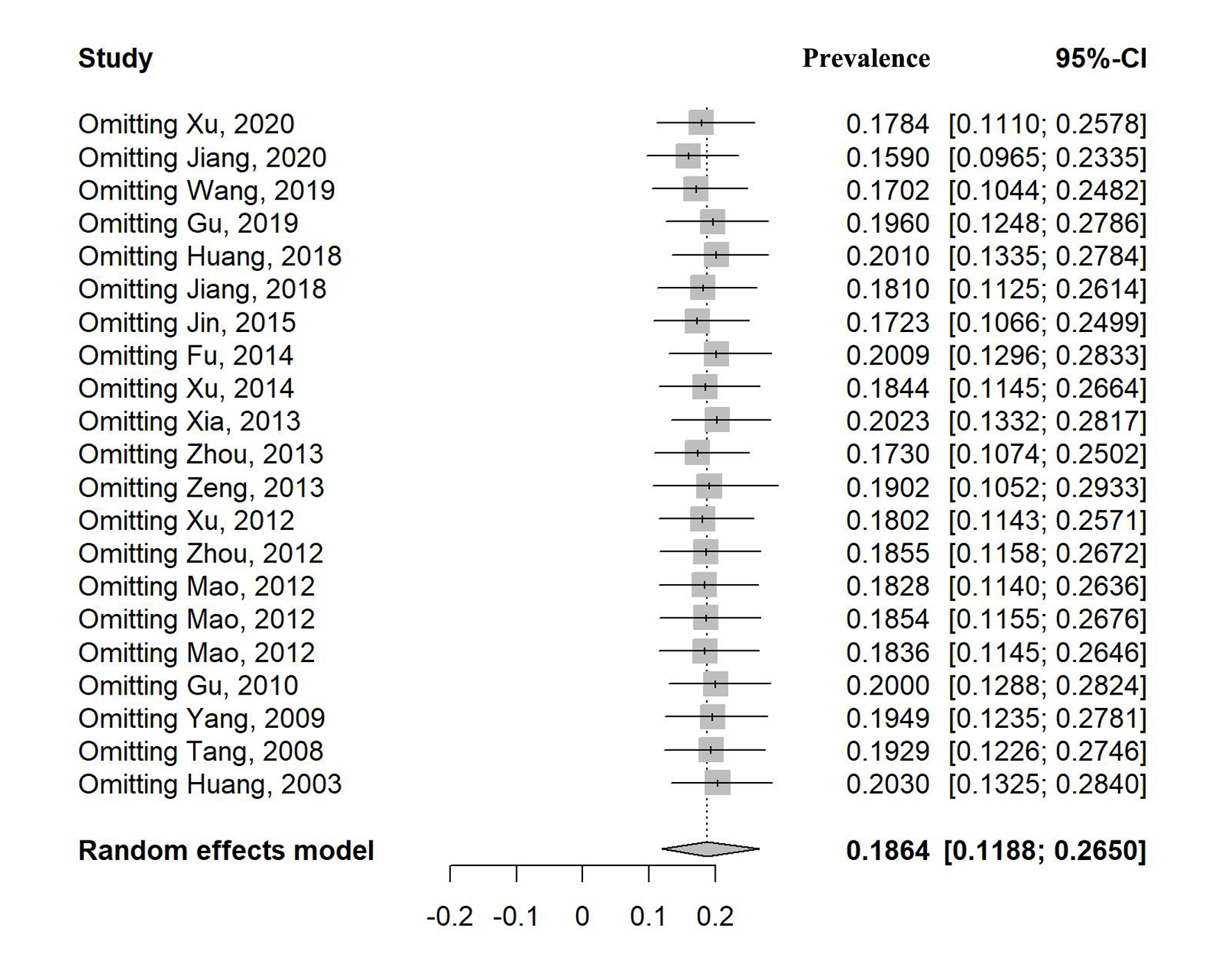

Supplement: Supplementary file 3 — Additional file 3: Fig. S1. Sensitivity analysis of the pooled prevalence of schistosomiasis liver abnormalities for all studies. [file 12879_2022_7241_MOESM3_ESM.zip › Additional file 3/Additional file 3_Fig. 1 The sensitivity analysis.tif]
